# Fucoidan Ameliorates Contrast-Induced Acute Kidney Injury in Mice by Modulating the TLR4/NF-κB and Nrf2/GPX4 Pathways

**DOI:** 10.3390/ph19081214

**Published:** 2026-08-01

**Authors:** Li Zhang, Qiaoling Zhao, Jing Tian, Fanghang Li, Yunping Tang, Yun Lu

**Affiliations:** 1 Medical Department, The Second Affiliated Hospital of Jiaxing University, Jiaxing 314000, China; jxeyzhangli@163.com; 2 Zhoushan Institute for Food and Drug Control, Zhoushan 316000, China; zql850410@126.com; 3 School of Food and Pharmacy, Zhejiang Ocean University, Zhoushan 316022, China; tianjing00326@163.com (J.T.); 13388796779@163.com (F.L.); tangyunping1985@163.com (Y.T.)

**Keywords:** fucoidan, contrast-induced acute kidney injury, oxidative stress, inflammation, ferroptosis, metabolomics

## Abstract

**Objectives:** The purpose of this study was to investigate the protective effects and underlying mechanisms of fucoidan on contrast-induced acute kidney injury (CI-AKI) in mice, focusing on the TLR4/NF-κB and Nrf2/GPX4 pathways. **Methods:** Five-week-old male ICR mice were randomly divided into normal control, contrast model, low-dose (100 mg/kg), and high-dose (300 mg/kg) fucoidan groups. Renal index, biochemical markers, histopathology, oxidative stress indicators, inflammatory cytokine levels, and the expression of TLR4/NF-κB, Nrf2/HO-1, and ferroptosis-related proteins were assessed. Untargeted metabolomics followed by KEGG pathway enrichment was also performed. **Results:** Our results showed that contrast successfully established the CI-AKI model, as evidenced by an increased kidney index, abnormal biochemical parameters, severe renal pathological damage, oxidative stress imbalance, inflammatory activation, ferroptosis, and metabolic disturbances. Fucoidan dose-dependently improved kidney index and biochemical markers, alleviated pathological injury, enhanced antioxidant capacity, suppressed inflammation and ferroptosis, and reversed metabolic pathway disorders (e.g., purine and glycerophospholipid metabolism), with the high dose showing more pronounced effects. **Conclusions:** Fucoidan could effectively ameliorate CI-AKI, and its effects are closely associated with the inhibition of the TLR4/NF-κB pathway, activation of the Nrf2/HO-1 pathway, regulation of ferroptosis-related proteins, and improvement of key metabolic disturbances, suggesting a new research direction for the prevention of CI-AKI.

## 1. Introduction

Iodine contrast media (ICM) are widely used in clinical invasive imaging examinations [1,2]. Following intravascular injection of ICM during diagnostic or interventional procedures, contrast-induced acute kidney injury (CI-AKI) represents a significant complication and has risen to be the third most frequent cause of hospital-acquired AKI [3,4]. CI-AKI prolongs hospitalization, negatively affects patient prognosis and recovery, increases in-hospital mortality, and adds to the healthcare burden [5,6]. Although intravenous and/or oral hydration before and after intra-arterial injection of ICM can be used to prevent CI-AKI, the effectiveness of current preventive and therapeutic strategies remains unsatisfactory, especially in elderly patients and those with hypertension, heart failure, or renal insufficiency, where their use is widely restricted [7,8]. Therefore, mitigating the nephrotoxicity induced by ICM has become an urgent clinical need [1,9].

Extensive clinical and experimental studies have confirmed that renal medullary hypoxia/ischemia, direct cytotoxicity of ICM, oxidative stress, and inflammatory responses are associated with the onset and progression of CI-AKI, which may result from the interplay of multiple mechanisms [1,9]. In recent years, multiple works have found that ferroptosis plays vital roles in various AKI models [10,11]. In CI-AKI, ICM downregulates glutathione peroxidase 4 (GPX4), a key ferroptosis regulator, leading to increased lipid peroxidation and tubular cell death [12,13]. Targeting ferroptosis has shown therapeutic promise: hemin, a clinical HO-1 inducer, activates the HO-1/Nrf2/GPX4 axis, reducing oxidative stress and renal injury in diabetic rats and HK-2 cells [14]. Similarly, the flavonoid icariin attenuates CI-AKI by suppressing oxidative stress, inhibiting NF-κB-driven inflammation, and blocking apoptosis [15]. Furthermore, a combination of the nano-antioxidant fullerenol and the vasodilator selexipag provides additive renoprotection by simultaneously scavenging free radicals and relieving intrarenal vasoconstriction [16]. These findings demonstrate that specifically intervening in ferroptosis, along with modulating oxidative stress and inflammation, can effectively ameliorate CI-AKI.

Fucoidan is a natural water-soluble sulfated heteropolysaccharide derived from brown seaweeds and some marine invertebrate tissues [17,18]. Its composition is complex, with sulfated α-L-fucose as the major component, along with small amounts of xylose, mannose, galactose, and uronic acids [19,20]. Studies have shown that fucoidan possesses anticancer, anti-inflammatory, antiviral, antioxidant, antithrombotic, and immunomodulatory activities [21,22,23]. However, there have been no reports on the use of fucoidan for the prevention of CI-AKI. Therefore, exploring the biological efficacy and mechanism of fucoidan in preventing CI-AKI holds profound significance and broad prospects. In this study, we established a mouse model of CI-AKI and, for the first time, systematically investigated the renoprotective effects of fucoidan by integrating classical pathway analysis (TLR4/NF-κB and Nrf2/GPX4) with untargeted kidney metabolomics. This integrative approach aims to not only confirm the involvement of known pathways but also to uncover novel metabolic signatures and pathway interactions that may underlie fucoidan’s multi-target protective effects in CI-AKI.

## 2. Results and Discussion

### 2.1. Effect of Fucoidan on Renal Function Indicators

**Figure 1:**
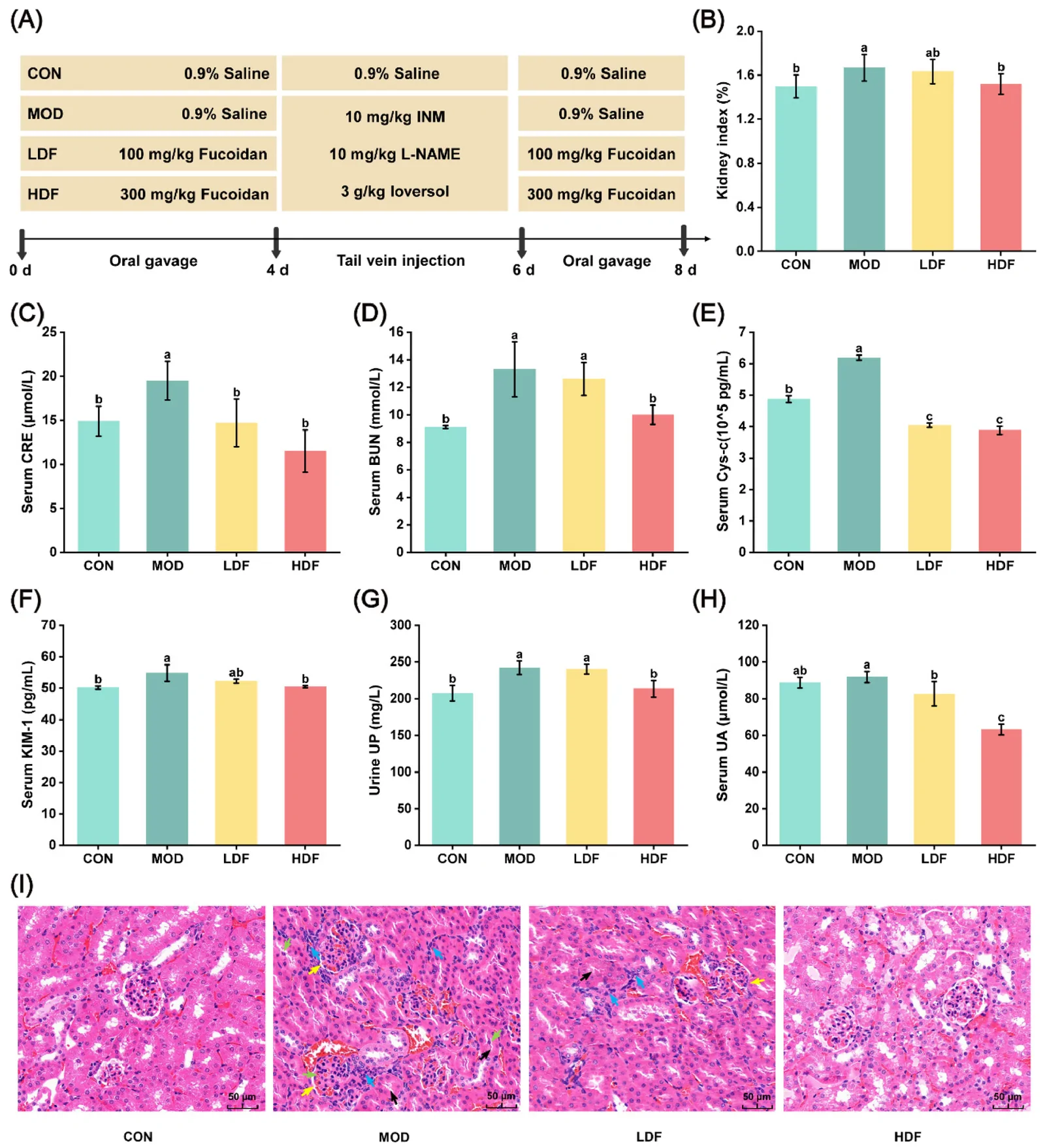
Effect of fucoidan on contrast-induced acute kidney injury in mice (*n* = 6). (**A**) Experimental design; (**B**) kidney index; (**C**) CRE; (**D**) BUN; (**E**) Cys-C; (**F**) KIM-1; (**G**) UP; (**H**) UA; (**I**) H&E staining (400 ×, 50 = μm). The yellow arrow indicates the narrowing of the Bowman’s capsule, the black arrow indicates the constriction of the proximal tubule lumen, the green arrow indicates sloughed-off cells, and the blue arrow indicates the infiltration of interstitial inflammatory cells. Different letters over bars indicate statistical significance between the two groups (*p* < 0.05); the same letter indicates no significant difference.

As shown in Figure 1A, mice were divided into four groups: normal control (CON), contrast model (MOD), low-dose fucoidan (LDF, 100 mg/kg), and high-dose fucoidan (HDF, 300 mg/kg). The kidney index is a key macroscopic indicator for evaluating renal edema and pathological hypertrophy [17]. Compared to the CON, the kidney index of the MOD group was significantly elevated (*p* < 0.05, Figure 1B), indicating marked renal swelling induced by the contrast. After fucoidan treatment, the kidney index of the HDF group decreased significantly compared to the MOD group (*p* < 0.05), and it was comparable to that of the CON, with no statistically significant difference. A decreasing trend was observed in the LDF group, but the difference relative to the MOD group was not statistically significant (*p* > 0.05). These results demonstrate that a high dose of fucoidan can effectively alleviate the macroscopic renal pathological changes induced by contrast.

CRE and BUN are traditional global markers of filtration function, while Cys-c offers a more stable and early estimate of glomerular filtration rate, and KIM-1 directly indicates tubular damage [24,25]. Their combination allows comprehensive assessment of both functional decline and structural renal injury. The results of serum renal function indicator tests (Figure 1C–F) showed that serum CRE, BUN, Cys-c, and KIM-1 levels in the MOD were significantly higher than those in the CON (*p* < 0.05), confirming the successful establishment of the CI-AKI model. After intervention with fucoidan, each indicator showed varying degrees of improvement. Specifically, the serum levels of CRE, Cys-c, and KIM-1 in the HDF group were significantly lower than those in the MOD (*p* < 0.05), and no notable difference was observed compared with the CON, supporting the restoration of renal homeostasis by HDF. In the LDF group, the above indicators also showed a decreasing trend, with CRE and Cys-c being significantly reduced (*p* < 0.05). Notably, for BUN, the HDF group was significantly lower than the MOD (*p* < 0.05), whereas the LDF group did not show a significant improvement.

Moreover, UP directly indicates glomerular-tubular damage, whereas UA reflects metabolic disturbances and oxidative stress that can aggravate injury. In our study, the UP excretion and serum UA levels in the MOD were significantly higher than those in the CON (Figure 1G,H, *p* < 0.05). After treatment with fucoidan, both UP and UA levels in the HDF group decreased significantly (*p* < 0.05). The LDF group showed a significant reduction in UA level (*p* < 0.05), while the decrease in UP level did not reach statistical significance. Overall, fucoidan significantly ameliorated contrast-induced abnormalities in serum and urine renal function indicators, and the ameliorative effect of the high-dose group was superior to that of the low-dose group, exhibiting a certain dose-dependent manner.

### 2.2. Fucoidan Ameliorates Renal Histopathological Injury

The HE staining results (Figure 1I) show that the renal tissue structure of mice in the CON group is normal, with no necrosis observed in the renal tubular epithelial cells and no inflammatory cell infiltration in the renal interstitium. In contrast, the kidneys of the MOD group mice exhibited severe pathological damage, with a noticeable reduction in the volume of some renal corpuscles and a significant narrowing of the Bowman’s capsule (indicated by the yellow arrows). The lumens of the proximal renal tubules were markedly constricted (indicated by the black arrows), and some renal tubules contained sloughed-off cells (indicated by the green arrows). Additionally, a considerable accumulation of inflammatory cells was observed infiltrating the renal interstitium (indicated by the blue arrows).

After intervention with fucoidan, the pathological damage in kidney tissue was significantly alleviated. In the LDF group mice, the morphological structure of the glomeruli and renal tubules showed some recovery, although some glomeruli remained small in volume, and a slight reduction in the lumen of some proximal tubules along with minor infiltration of inflammatory cells was observed. The kidney tissue morphology in the HDF group mice exhibited even more significant improvement, with the structures of the glomeruli and Bowman’s capsule approaching those of the CON group, and no sloughed-off cells were found within the lumens of the renal tubules. These results indicate that fucoidan can significantly reduce iodine-induced pathological damage in kidney tissue and facilitate the repair of renal tissue structure.

### 2.3. Fucoidan Regulates Renal Oxidative Stress Levels

**Figure 2:**
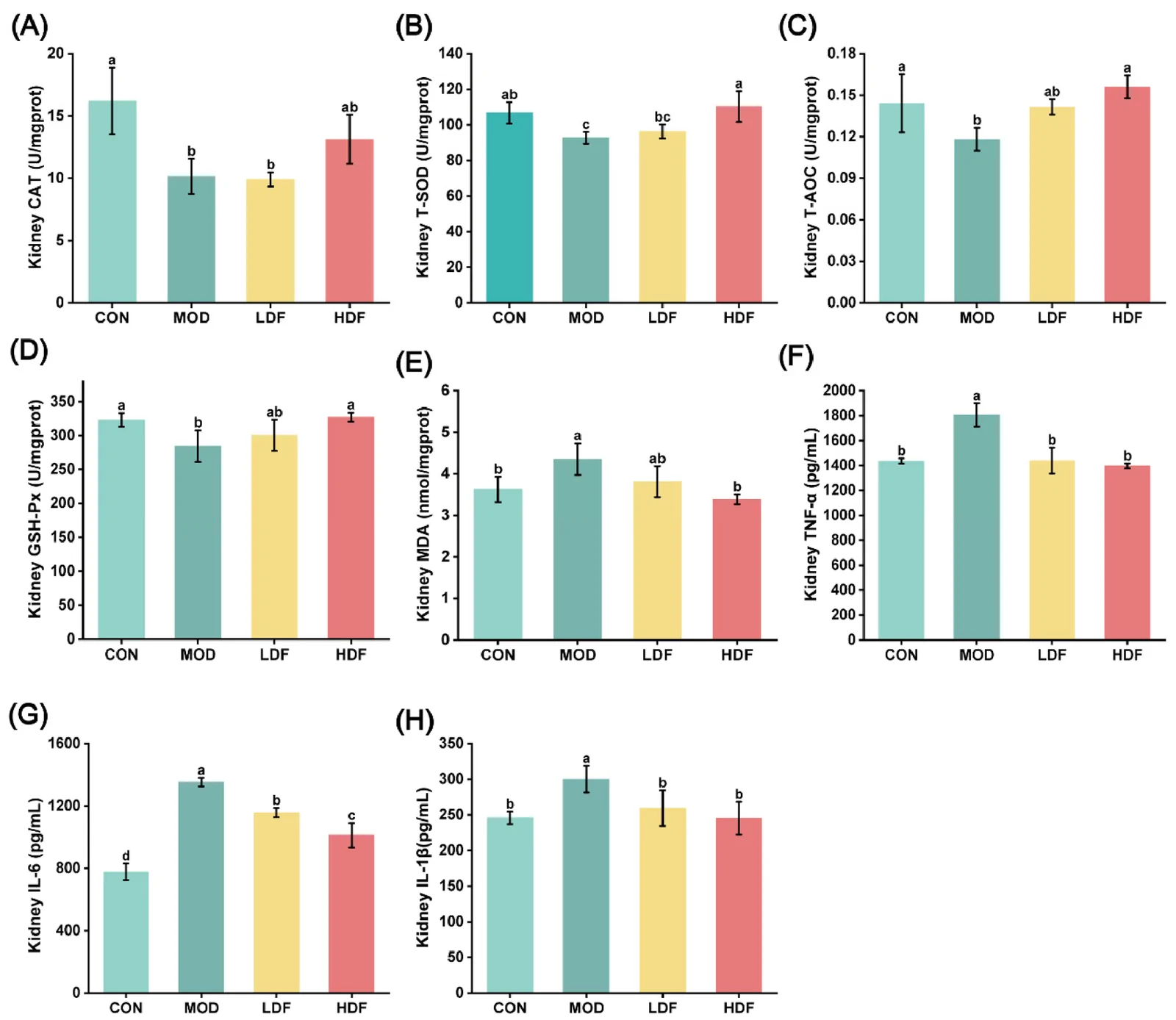
The effects of fucoidan treatment on oxidative stress and inflammation caused by contrast. (**A**) CAT; (**B**) T-SOD; (**C**) T-AOC; (**D**) GSH-Px; (**E**) MDA; (**F**) TNF-α; (**G**) IL-6; (**H**) IL-1β. The same letter indicates no significant difference.

Oxidative stress plays a key role in the pathogenesis of CI-AKI [26,27]. Previous research has shown that fucoidan protects the kidneys primarily by restoring redox balance [28]. Moreover, numerous studies have confirmed its antioxidant properties, including scavenging free radicals and enhancing the endogenous antioxidant enzyme activities [29,30]. To investigate whether fucoidan exerts renal protective effects by modulating oxidative stress, we measured antioxidant enzyme activities and oxidative damage product levels in kidney tissues (Figure 2A–E). Compared with the CON, the T-SOD, CAT, GSH-Px activities, and T-AOC in kidney tissues of the MOD were significantly decreased (*p* < 0.05), indicating that contrast induction severely impaired the renal antioxidant defense system. After fucoidan intervention, these antioxidant parameters recovered to varying degrees. Specifically, the T-SOD, CAT, and GSH-Px activities, and T-AOC in the HDF group were significantly higher than those in the MOD (*p* < 0.05) and were not significantly different from those in the CON. The LDF group also showed significant increases (*p* < 0.05), but the recovery was less pronounced than that in the HDF group. These results indicate that fucoidan effectively enhances renal antioxidant enzyme activities and increases tissue antioxidant capacity in a dose-dependent manner.

Regarding oxidative damage products (Figure 2E), the renal MDA content in the MOD was significantly higher than that in the CON (*p* < 0.05), suggesting that contrast induction caused severe lipid peroxidation injury. After fucoidan administration, MDA content dropped substantially, with a stronger reduction in the HDF group (*p* < 0.05) and a significant decreasing trend in the LDF group (*p* < 0.05). Overall, these data demonstrate that fucoidan ameliorates contrast-induced renal oxidative stress injury by strengthening the antioxidant defense machinery and reducing the accumulation of oxidative damage products, corroborating previous findings [28].

### 2.4. Fucoidan Inhibits the Renal Inflammatory Response

The progression of CI-AKI is critically driven by excessive activation of the inflammatory response [31]. Compared with the CON (Figure 2F–H), the TNF-α, IL-6, and IL-1β levels in renal tissues of the MOD group were significantly increased (*p* < 0.05), showing that contrast induced a severe renal inflammatory response, which was consistent with previous studies [32,33]. After fucoidan intervention, the levels of the above pro-inflammatory cytokines were significantly reduced in both the LDF and HDF groups (*p* < 0.05), with the HDF group showing a greater reduction. These results demonstrate that fucoidan inhibits contrast-induced renal inflammation in a dose-dependent manner and alleviates inflammation-mediated structural and functional injury to the kidney.

### 2.5. Effects of Fucoidan on the Profile of Kidney Metabolites

**Figure 3:**
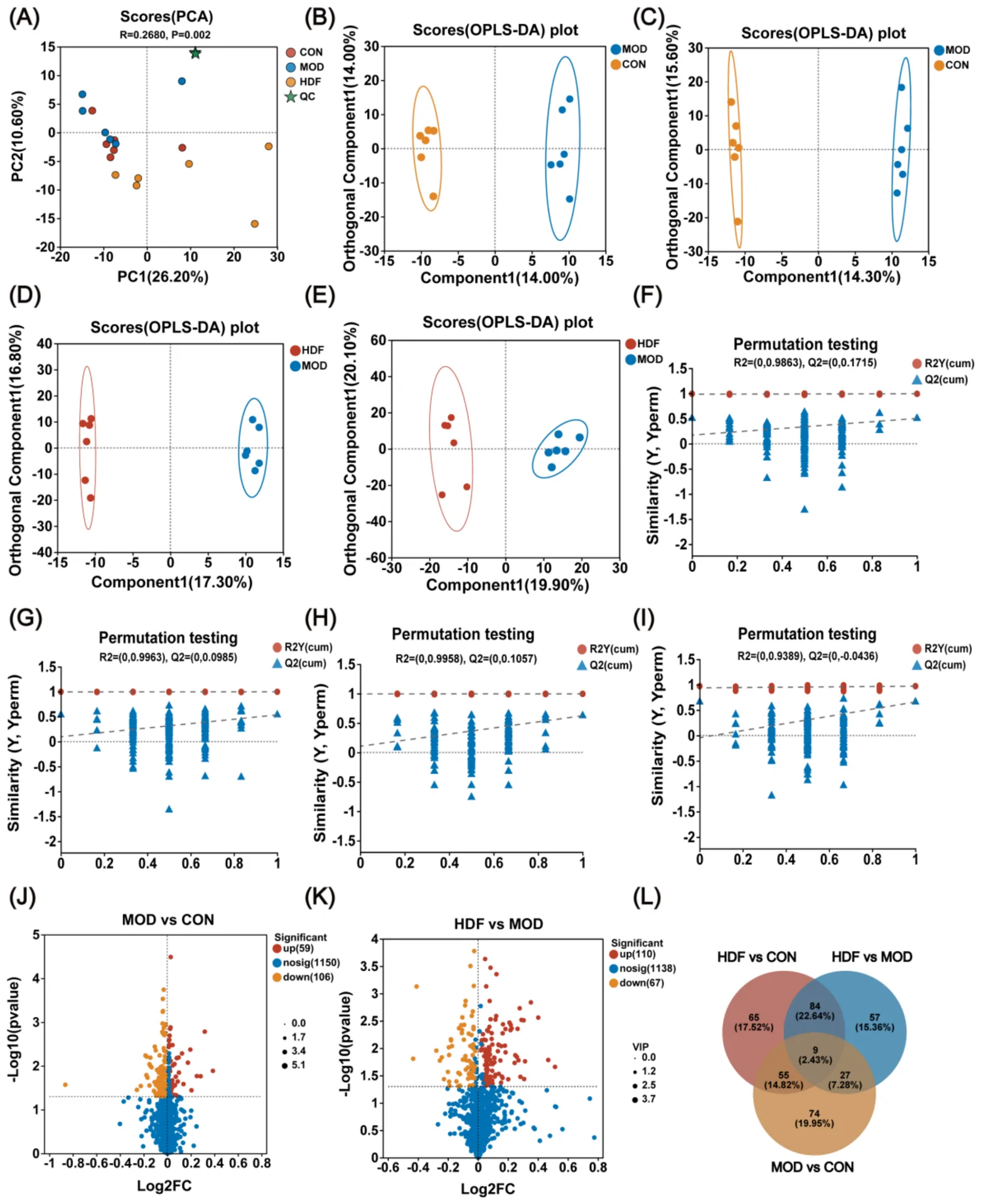
Effects of fucoidan on renal metabolic profiles and differential metabolites in CI-AKI mice: (**A**) PCA score plot; (**B**–**E**) OPLS-DA score plots; (**F**–**I**) permutation test plots corresponding to the OPLS-DA models; (**J**) volcano plot of differential metabolites in MOD vs. CON group; (**K**) volcano plot of differential metabolites in HDF vs. MOD group; (**L**) Venn diagram of differential metabolites among groups. Note: Differential metabolites were identified using criteria of VIP > 1 and *p* < 0.05.

Untargeted metabolomics is widely used for early disease screening, biomarker discovery, and mechanistic research [34]. Comparing metabolic profiles across different groups enables the identification of differential metabolites and key dysregulated pathways, providing a global metabolic perspective to reveal the pathological basis of diseases and discover potential intervention targets [35,36]. In this study, PCA was first conducted to evaluate global metabolic differences among groups and to assess analytical stability (Figure 3A). The PCA score plot revealed distinct clustering of the CON, MOD, and HDF groups along with the quality control (QC) samples. The first two principal components explained 36.9% of the total metabolic variance (PC1 = 26.2%, PC2 = 10.7%), and samples from the CON, MOD, and HDF groups exhibited a clear separation trend on the PCA score plot. Analysis of similarities (ANOSIM) was further performed to verify the statistical significance of group separation, yielding R = 0.2680 and *p* = 0.002. This result indicated that the inter-group metabolic difference was significantly greater than the intra-group random variation, confirming that contrast exposure and fucoidan treatment significantly remodeled the renal metabolic profile of mice. To further identify differential metabolites between groups, an OPLS-DA model was established. As shown in Figure 3B–E, the OPLS-DA score plots for the MOD vs. CON, HDF vs. MOD, and HDF vs. CON comparisons all exhibited pronounced group separation. Permutation tests (Figure 3F–I) demonstrated that for each OPLS-DA model, the R^2^Y (cum) and Q^2^ (cum) values were significantly higher than those of the permuted models, with the intercepts of Q^2^ (cum) close to zero (range 0.0436–0.1715), confirming that the models were stable, reliable, and free from overfitting, thus suitable for subsequent differential metabolite screening.

Based on volcano plot analysis (Figure 3J,K) and using the criteria of VIP > 1.0 and *p* < 0.05, a total of 165 significantly differential metabolites were identified in the MOD vs. CON comparison (Figure 3J), of which 59 were upregulated and 106 downregulated. In the HDF vs. MOD comparison (Figure 3K), 177 significantly differential metabolites were identified, including 110 upregulated and 67 downregulated. Venn diagram analysis (Figure 3L) revealed that nine common differential metabolites (accounting for 2.43% of the total) overlapped between the MOD vs. CON and HDF vs. MOD comparisons, suggesting that these metabolites represent core metabolic targets involved in CI-AKI and fucoidan intervention.

**Figure 4:**
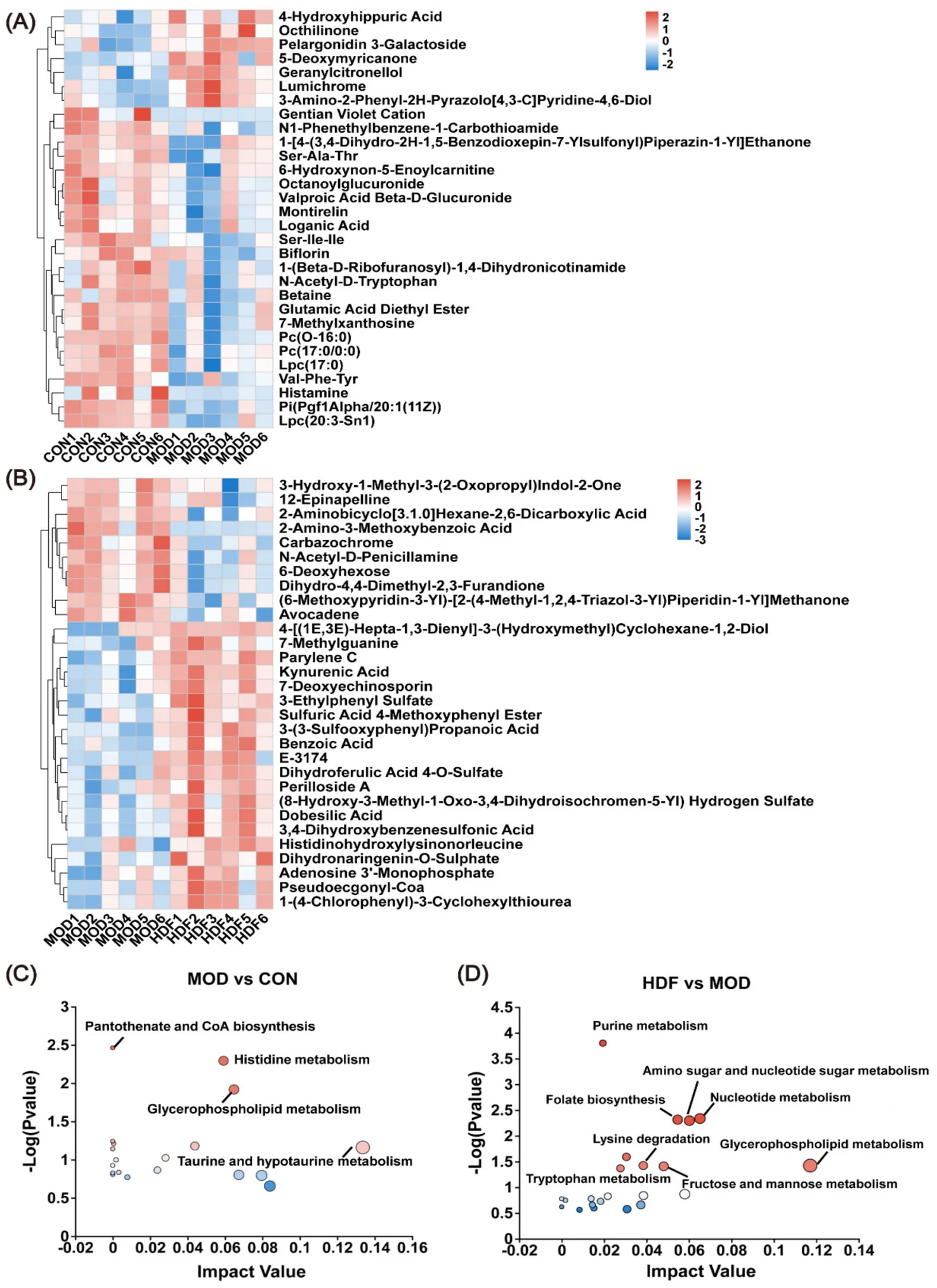
Effects of fucoidan on differential metabolites and enriched pathways in kidneys of CI-AKI mice: (**A**) Hierarchical clustering heatmap of differential metabolites in the MOD vs. CON group; (**B**) hierarchical clustering heatmap of differential metabolites in the HDF vs. MOD group; (**C**) KEGG pathway enrichment bubble plot of differential metabolites in the MOD vs. CON group; (**D**) KEGG pathway enrichment bubble plot of differential metabolites in the HDF vs. MOD group.

Hierarchical clustering heatmaps (Figure 4A,B) revealed distinct group-specific metabolic profiles, with samples from the CON, MOD, and HDF groups clustering separately, confirming characteristic metabolic alterations induced by contrast and fucoidan. KEGG pathway enrichment analysis (Figure 4C,D) showed that in the MOD vs. CON comparison, differential metabolites were primarily enriched in pantothenate and CoA biosynthesis, histidine metabolism, glycerophospholipid metabolism, and taurine and hypotaurine metabolism, indicating that disruption of energy, amino acid, and lipid metabolism underlies CI-AKI. In contrast, in the HDF vs. MOD comparison, differential metabolites were significantly enriched in purine metabolism, amino sugar and nucleotide sugar metabolism, nucleotide metabolism, folate biosynthesis, and glycerophospholipid metabolism. Among the identified pathways, purine metabolism exhibited the most significant enrichment in the fucoidan-treated group. This finding is mechanistically relevant, as purine catabolism via xanthine oxidase generates both uric acid and reactive oxygen species (ROS), which are known to activate the NLRP3 inflammasome and drive NF-κB-mediated inflammation [37]. Accordingly, the reversal of purine metabolic disturbances by fucoidan likely contributes to the suppression of TLR4/NF-κB signaling observed in our study. Furthermore, imbalances in nucleotide metabolism can impair the GSH-dependent antioxidant defense system and disrupt intracellular redox homeostasis [38]. Thus, the restoration of this pathway by fucoidan may reinforce Nrf2/HO-1-mediated antioxidant defense and mitigate inflammatory injury. Glycerophospholipid metabolism, another major pathway affected, is closely linked to ferroptosis. Glycerophospholipids are the primary source of polyunsaturated fatty acids (PUFAs) in membrane phospholipids; their oxidation under iron-catalyzed conditions produces lipid peroxides that drive ferroptosis when GPX4 activity is compromised [39]. Our findings suggest that fucoidan restores renal metabolic homeostasis by counteracting contrast-induced perturbations, particularly through modulation of purine, nucleotide, and glycerophospholipid metabolism.

### 2.6. Effect of Fucoidan on the TLR4/NF-κB Pathway

**Figure 5:**
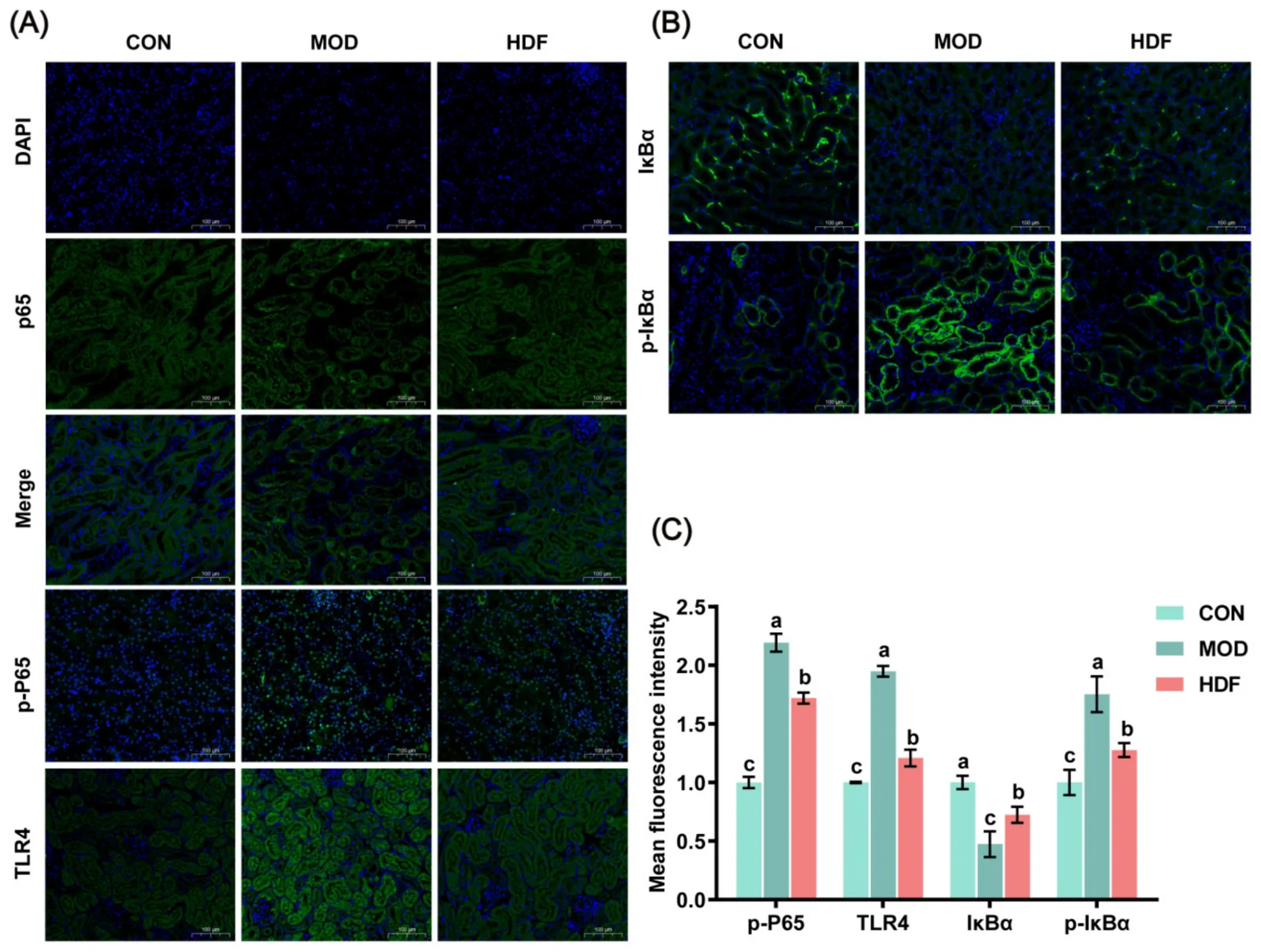
Immunofluorescence staining analysis of the effect of fucoidan on the TLR4/NF-κB pathway (*n* = 3). (**A**) Immunofluorescence images showing p65, p-P65, and TLR4 protein (scale bar = 100 μm); (**B**) immunofluorescence images showing IκBα and p-IκBα (scale bar = 100 μm); (**C**) the quantification of mean fluorescence intensity regarding p-P65, TLR4, IκBα, and p-IκBα. The same letter indicates no significant difference.

The TLR4/NF-κB pathway is a classical mediator of inflammatory responses [34]. TLR4 recognizes various danger signals and triggers downstream activation of NF-κB [24]. Under resting conditions, P65 is sequestered in the cytoplasm by binding to its inhibitor IκBα. Upon pathway activation, IκBα is phosphorylated (p-IκBα) and degraded, allowing P65 to be phosphorylated (p-P65) and translocate into the nucleus to drive pro-inflammatory gene expression [24]. As shown by immunofluorescence results (Figure 5), compared with the CON group, the mean fluorescence intensities of TLR4, p-P65, and p-IκBα in the kidney tissues of MOD group mice were significantly increased (*p* < 0.05), whereas the mean fluorescence intensity of IκBα was significantly decreased (*p* < 0.05). These findings indicate that contrast-induced injury can robustly activate the TLR4/NF-κB pathway and promote downstream inflammatory signaling. After intervention with high-dose fucoidan, the fluorescence intensities of TLR4, p-P65, and p-IκBα were all significantly downregulated (*p* < 0.05), while the fluorescence intensity of IκBα was significantly upregulated (*p* < 0.05). This suggests that fucoidan effectively suppresses the excessive activation of the TLR4/NF-κB pathway, thereby blocking the inflammatory cascade and alleviating renal inflammatory injury. These findings corroborate those of Tian et al. [17], suggesting that fucoidan has the potential to ameliorate CTX-induced kidney injury by regulating the TLR4/NF-κB pathway.

### 2.7. Effect of Fucoidan on the Nrf2/GPX4 Axis

**Figure 6:**
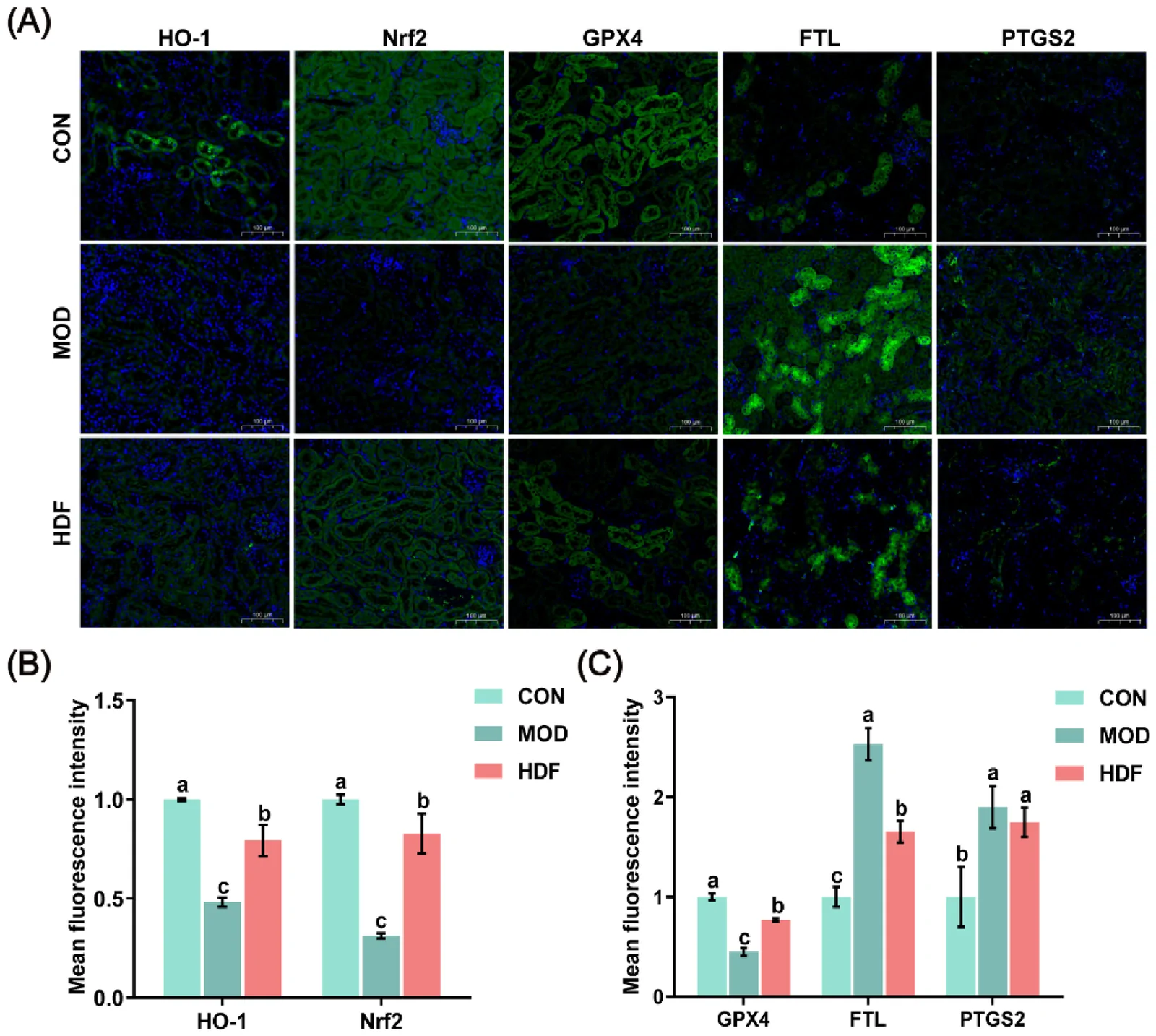
Immunofluorescence staining analysis of the effect of fucoidan on the Nrf2/GPX4 axis (*n* = 3). (**A**) Immunofluorescence images showing HO-1, Nrf2, GPX4, FTL, and PTGS2 (scale bar = 100 μm); (**B**) the quantification of mean fluorescence intensity regarding HO-1 and Nrf2; (**C**) the quantification of mean fluorescence intensity regarding GPX4, FTL, and PTGS2. The same letter indicates no significant difference.

The Nrf2/HO-1 axis serves as a key endogenous defense mechanism against oxidative stress in the body [40]. Under normal conditions, Nrf2 remains inactive by binding to the cytoplasmic protein Keap1. When oxidative stimuli occur, Nrf2 separates from Keap1 and moves into the nucleus. Once inside, it binds to the antioxidant response element (ARE), triggering the transcription of various downstream antioxidant enzymes and phase II detoxifying enzymes, such as HO-1. This cascade helps scavenge reactive oxygen species and reduce lipid peroxidation injury [41,42]. As indicated in Figure 6A,B, compared with the CON, the mean fluorescence intensities of Nrf2 and its downstream target protein HO-1 in the kidney tissues of MOD group mice were significantly decreased (*p* < 0.05), indicating that contrast-induced injury inhibited the activation of the Nrf2/HO-1 pathway and weakened the renal antioxidant defense capacity. After intervention with fucoidan, the fluorescence intensities of Nrf2 and HO-1 were significantly increased (*p* < 0.05). These findings demonstrate that fucoidan enhances the endogenous antioxidant capacity of the kidney by activating the Nrf2/HO-1 pathway, which corroborates the improvement in oxidative stress indicators described above, further supporting that the protective effect of fucoidan against CI-AKI is closely associated with the reactivation of this pathway. Huang et al. [19] reported that fucoidan ameliorates renal injury in diabetic nephropathy rats through activation of the PI3K/AKT/Nrf2 pathway, and Tian et al. [17] also demonstrated that fucoidan alleviates CTX-induced kidney injury by regulating the Nrf2/HO-1 pathway, all of which are consistent with our current findings.

Ferroptosis is an important mode of contrast-induced injury to renal tubular epithelial cells [14,43]. In this study, we further examined the expression of core regulatory proteins involved in ferroptosis. Immunofluorescence results (Figure 6A,C) showed that, compared with the CON, the mean fluorescence intensity of GPX4 in the kidney tissues of MOD group mice was significantly decreased (*p* < 0.05), while FTL and PTGS2 were significantly increased (*p* < 0.05), indicating that contrast-induced injury induced ferroptosis in the kidney. After intervention with fucoidan, the fluorescence intensity of GPX4 was significantly upregulated (*p* < 0.05), and that of FTL was significantly downregulated (*p* < 0.05). However, no significant difference was observed in the fluorescence intensity of PTGS2 compared with the MOD (*p* > 0.05). These results indicate that fucoidan suppresses contrast-induced renal ferroptosis through modulation of key ferroptosis-associated proteins, including GPX4 and FTL, thus preserving both the structural and functional integrity of renal tubular epithelial cells. Consistent with our results, previous studies have shown that fucoidan exerts anti-ferroptotic effects in other pathological contexts. Wang et al. [44] reported that fucoidan alleviates doxorubicin-induced cardiotoxicity by inhibiting ferroptosis via the Nrf2/GPX4 pathway, while Li et al. [45] demonstrated that fucoidan ameliorates ferroptosis in ischemia–reperfusion-induced liver injury through activation of the Nrf2/HO-1/GPX4 axis. Together with these reports, our study further supports that fucoidan’s protective effect against CI-AKI is mediated, at least in part, by suppressing ferroptosis via Nrf2-related pathways.

### 2.8. Correlation Analysis of Differential Indicators

**Figure 7:**
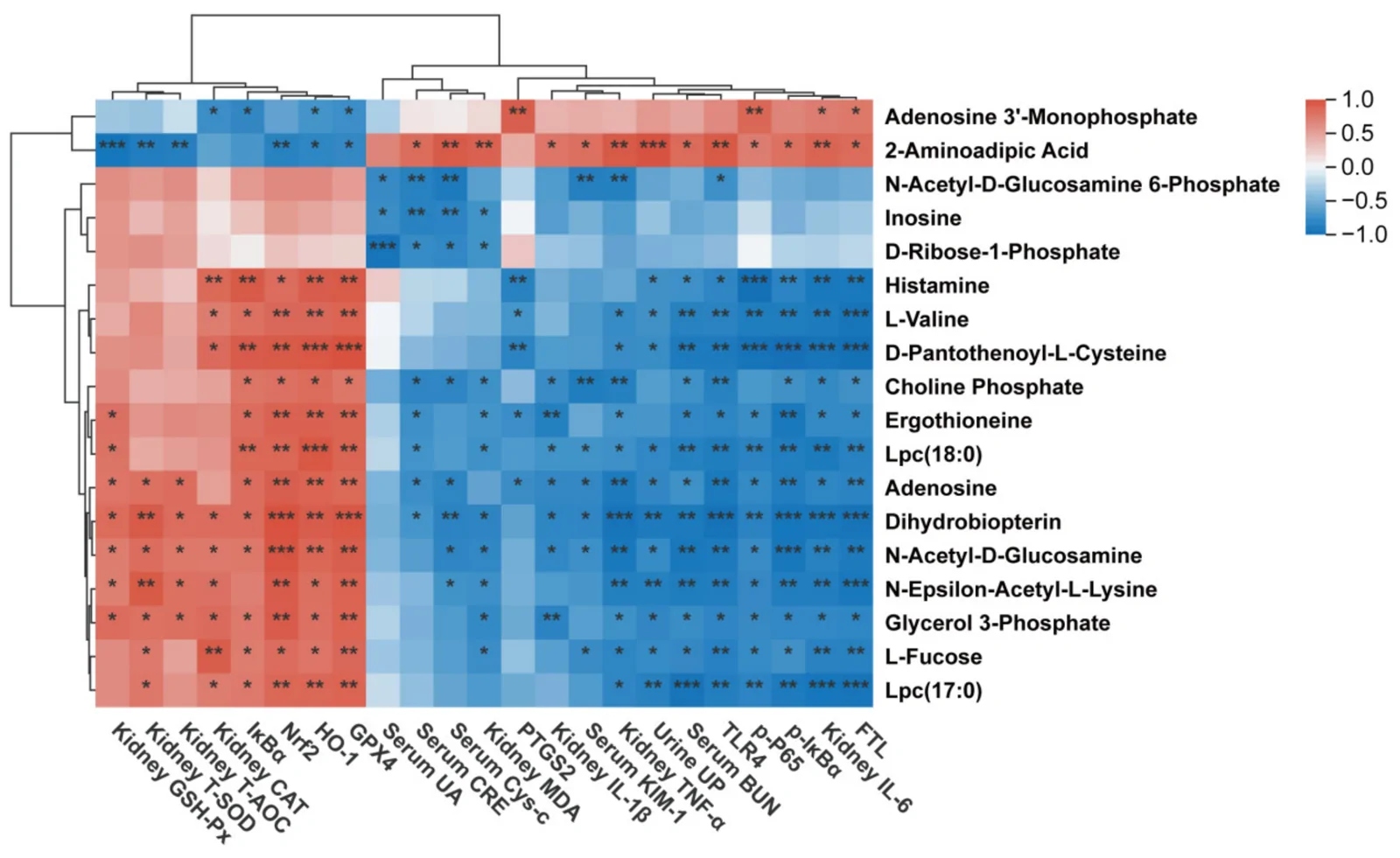
Correlation analysis between metabolites and biochemical indicators. In this analysis, red indicates a positive correlation, while blue indicates a negative correlation; the larger the circle and the darker the color, the stronger the correlation (* *p* < 0.05, ** *p* < 0.01, *** *p* < 0.001).

Using a screening threshold of |r| > 0.8 and *p* < 0.05, Pearson correlation coefficient analysis was conducted to further elucidate the intrinsic relationships between differentially expressed metabolites within important pathways, biochemical indicators, and associated proteins. As illustrated in Figure 7, the differentially metabolized compounds histamine, L-v, D-pantothenoyl-L-cysteine, Lpc (18:0), adenosine, dihydrobiopterin, N-acetyl-D-glucosamine, N-epsilon-acetyl-L-lysine, glycerol 3-phosphate, L-fucose, and Lpc (17:0) were significantly negatively correlated with proteins FTL, p-IκBα, p-P65, TLR4, kidney IL-6, kidney TNF-α, serum BUN, and urine UP, while showing a positive correlation with GPX4, HO-1, Nrf2, IκBα, kidney T-AOC, and renal antioxidant enzymes (CAT, T-SOD, and GSH-Px). However, adenosine 3′-monophosphate and 2-aminoadipic acid exhibited the opposite correlation pattern. This finding confirms that fucoidan plays a crucial role in regulating renal metabolic disorders induced by ICM and improving changes in biochemical indicators.

### 2.9. Potential Translational Perspective

Beyond these mechanistic insights, it is important to consider the translational relevance of our findings. Fucoidan has been evaluated in multiple human clinical trials across diverse indications, with a consistent safety profile. A randomized, double-blind, placebo-controlled trial in patients with type 2 diabetes administered a daily 60 mL beverage containing 1.62 g of high-molecular-weight fucoidan for 12 weeks, and no adverse events occurred during the study period [46]. Similarly, a pilot study in healthy adults ingesting 3.0 g/day of Okinawa mozuku-derived fucoidan for 12 weeks reported no clinically adverse events [47]. Furthermore, a safety evaluation of excessive ingestion demonstrated that taking mozuku fucoidan at doses up to 4.05 g daily for two weeks caused no abnormalities in abdominal symptoms, fecal status, blood, or urine parameters [48]. Collectively, these cumulative data indicate that fucoidan has a well-established safety profile in humans across a wide dose range.

Importantly, the dose used in our HDF group (300 mg/kg in mice), when converted to a human equivalent dose using standard body surface area normalization, corresponds to approximately 1.464 g daily for a 60 kg adult. This value is strikingly close to the 1.62 g daily dose already tested in the type 2 diabetes trial and falls well within the range of doses (1.62 g to 4.05 g daily) that have been proven safe in other human studies. The dose-dependent efficacy observed in our study, with 300 mg/kg showing significantly greater renoprotection than 100 mg/kg, further supports the biological relevance of this dose range. Critically, these convergent findings indicate that the evaluated dose regimen is not only pharmacologically rational but, more importantly, clinically achievable based on existing human safety data. Consequently, this dose conversion primarily supports the feasibility of initial dose selection and safety margin assessment in future clinical translation, providing a practical rationale for designing well-controlled human trials to investigate fucoidan as an adjunctive strategy for CI-AKI prevention. Nevertheless, the definitive clinical efficacy must ultimately be established through properly designed, dose-escalation, randomized controlled trials in the target patient population.

## 3. Materials and Methods

### 3.1. Materials and Reagents

Fucoidan, obtained from Shandong Jiejing Group Co., Ltd. (Rizhao, China), has an average molecular weight of 250 kDa and consists of mannose, rhamnose, galactose, xylose, and fucose in a molar ratio of 2.04: 0.58: 1.04: 3.91: 12.43 [17]. Iopromide injection was provided by The Second Affiliated Hospital of Jiaxing University. Indomethacin (INM) and N’-nitro-L-arginine methyl ester hydrochloride (L-NAME) were purchased from Macklin (Shanghai, China). Assay kits for CRE, BUN, UA, UP, T-SOD, CAT, GSH-Px, MDA, and T-AOC were obtained from Jiancheng (Nanjing, China). ELISA kits for Cys-c, KIM-1, IL-1β, IL-6, and TNF-α were sourced from Elabscience (Wuhan, China). Primary antibodies against Nrf2 (AF0639) and HO-1 (AF5393) were purchased from Affinity Biosciences (Liyang, China). Primary antibodies against IκBα (66418-1-Ig), p-IκBα (82349-1-RR), GPX4 (67763-1-Ig), PTGS2 (66351-1-Ig), and FTL (10727-1-AP) were acquired from Proteintech Group (Wuhan, China). Primary antibodies against p-NF-κB p65 (AF5875), TLR4 (AF8187), and NF-κB p65 (AF0246) were provided by Beyotime Biotechnology (Shanghai, China).

### 3.2. Animal Experiments

Male ICR mice (5 weeks, 20 ± 2 g) were obtained from Hangzhou Muhao Biotechnology Co., Ltd., and all animal procedures were approved by the Animal Ethics Committee of Jiaxing University (No. JUMC2024-122) on April 16, 2024. All mice were acclimated in an SPF-level animal facility (temperature 23 ± 2 °C, humidity 60 ± 5%, 12 h light/dark cycle) for one week and then randomly divided into four groups (Figure 1A, *n* = 6): CON, MOD, LDF (100 mg/kg fucoidan), and HDF (300 mg/kg fucoidan). The total experimental period lasted 15 days, which included 7 days for adaptive feeding, followed by 8 days for both model induction and treatment administration. The LDF and HDF treatment groups were gavaged with 100 mg/kg and 300 mg/kg of fucoidan daily, respectively, while the CON and MOD groups received an equivalent volume of saline. After the administration on the fourth day, all mice were fasted and deprived of water for 12 h. Subsequently, mice in the LDF, HDF, and MOD groups were given a tail vein injection of indomethacin (10 mg/kg), followed 15 min later by a tail vein injection of L-NMMA (10 mg/kg), and 15 min after that, a tail vein injection of iopromide injection (3 g/kg) to induce kidney injury for 1 day [15,49]. The CON group was administered an equivalent amount of saline via tail vein injection. On the final day of dosing, the urine of the mice was collected. Finally, after 12 h of fasting but with water allowed, the mice were weighed, and blood was collected from the eyeball. They were then euthanized by decapitation, and the kidneys were immediately removed, rinsed with saline, and processed for further analysis. The kidney index was calculated as follows: kidney index (%) = kidney weight (g)/body weight (g) × 100%.

### 3.3. Determination of Biochemical Indicators

Blood from the eyeball was first coagulated at room temperature for 30–60 min and subsequently centrifuged at 3500× *g* for 10 min at 4 °C to yield the serum. Serum levels of UA, CRE, BUN, Cys-c, and KIM-1, as well as urinary protein content, were measured following the instructions, respectively. Kidney tissues were homogenized in pre-chilled saline using a handheld homogenizer, with a tissue weight (g) to saline volume (mL) ratio of 1:9, to prepare a 10% (*w*/*v*) homogenate. The homogenate was then centrifuged at 13,000× *g* for 10 min to obtain the supernatant. Protein concentration in the supernatant was determined using a BCA kit. Subsequently, the levels of antioxidant enzymes (CAT, T-SOD, T-AOC, and GSH-Px), MDA, and inflammatory cytokines (IL-1β, IL-6, and TNF-α) in the kidney homogenate were measured according to the corresponding kit instructions.

### 3.4. Histopathological Analysis

Kidney samples were fixed in 4% paraformaldehyde for 24 h, followed by paraffin embedding. Sections of 5-μm thickness were then prepared, deparaffinized, and subjected to H&E staining [24]. Images were taken under a CX31 light microscope (Olympus, Tokyo, Japan).

### 3.5. Metabolomics Analysis

Kidney samples (50 mg) from the CON, MOD, and HDF mouse groups were subjected to non-targeted metabolomics analysis by Shanghai Meiji Biopharmaceutical Technology Co., Ltd. As part of the system conditioning and quality control process, a pooled quality control sample (QC) was prepared by mixing equal volumes of all samples. The QC samples were disposed of and tested in the same manner as the analytic samples. It helped to represent the whole sample set, which would be injected at regular intervals (every 5–15 samples) in order to monitor the stability of the analysis. A pooled QC sample, prepared by mixing equal aliquots of all samples, was inserted regularly throughout the analytical run to monitor system stability. Features with relative standard deviation (RSD) > 30% in QC samples were removed to ensure data quality, and QC sample clustering was confirmed by principal component analysis (PCA). Public databases such as HMDB and Metlin were used for metabolite identification and annotation. The metabolite data were then uploaded to the Meiji Cloud platform for subsequent processing. To assess model stability, PCA, OPLS-DA, and permutation tests were performed. A threshold of VIP > 1 and *p* < 0.05 was used to select differential metabolites, which were then submitted to KEGG enrichment analysis to pinpoint key metabolic pathways [50].

### 3.6. Immunofluorescence Analysis

For immunofluorescence staining, primary antibodies were applied to paraffin-embedded kidney sections and incubated overnight at 4 °C. After washing, the sections were covered with matching secondary antibodies and incubated in the dark for 50 min at room temperature. Nuclei were then counterstained with DAPI, and an agent for quenching autofluorescence was added to reduce tissue-derived background signals. The slides were subsequently sealed with an anti-fluorescence quenching mounting medium and photographed under a microscope. Images were first captured using CaseViewer software 2.4.0.119028. For quantitative analysis, the acquired TIFF images were imported into ImageJ software 1.52i. The images were converted to 8-bit format, and channel splitting was performed. Using the threshold segmentation method, we set the “Threshold” to separate the target fluorescence signals and define regions of interest (ROIs), while excluding background interference. The average fluorescence intensity of each ROI was then measured for subsequent statistical analysis.

### 3.7. Statistical Analysis

Graphical plotting and statistical analysis of experimental data were performed using Origin 2021 and SPSS 27.0 software. Before performing the one-way analysis of variance (ANOVA), the normality of the data was assessed using the Shapiro–Wilk test, and the homogeneity of variances was evaluated using Levene’s test. The results confirmed that the data met the assumptions of normality (*p* > 0.05) and homogeneity of variance (*p* > 0.05) for all groups. Intergroup differences were tested using ANOVA followed by the LSD post hoc test. The experimental results are expressed as the mean ± standard deviation (SD), and *p* < 0.05 was considered statistically significant.

## 4. Conclusions

In summary, the dose-dependent renoprotective effect against CI-AKI observed in mice is associated with fucoidan treatment. Mechanistically, our findings suggest that fucoidan’s protective effects are closely associated with a multi-target modulation, including activation of the Nrf2/HO-1 antioxidant pathway, inhibition of the TLR4/NF-κB inflammatory cascade, regulation of ferroptosis-related proteins, and amelioration of disturbed purine, glycerophospholipid, and histidine metabolism. These findings provide a promising candidate for CI-AKI prevention and lay an experimental foundation for further mechanistic studies. However, several limitations should be acknowledged: (1) the metabolomic data are hypothesis-generating, and the putative roles of both the identified metabolites (adenosine, L-fucose, lysophosphatidylcholines, etc.) and the TLR4/NF-κB and Nrf2/GPX4 pathways remain to be causally proven; (2) the limited oral bioavailability of high-molecular-weight fucoidan may affect clinical translation; (3) our acute model in healthy mice does not fully replicate the complex comorbidity profiles of clinical patients. Future studies are indispensable to clarify the causal links and relative pathway contributions. These should integrate multiple complementary approaches, including: (1) enzyme-selective or pathway-specific inhibitors and genetic manipulation (silencing or overexpression) to establish causality; (2) targeted analytical approaches to independently validate the metabolomic findings; and (3) quantitative protein assays such as Western blotting to corroborate the immunofluorescence data and provide reliable protein-level evidence. In addition, pharmacokinetic and efficacy evaluations in comorbid or emergency settings, along with sex-balanced designs and larger sample sizes, are needed before clinical application.

## Data Availability

Data is contained within the article.

## References

[1] Li Y.Y., Wang J.D. (2024). Contrast-induced acute kidney injury: A review of definition, pathogenesis, risk factors, prevention and treatment. BMC Nephrol..

[2] Mohamed N.S.A., Alkharji F.W.A., Ghareeb M.F., Aljabr A. (2025). Risk of acute kidney injury following intravenous iodinated contrast exposure among pediatric population: A narrative review. Pediatr. Radiol..

[3] Su B.W., Yang H.X., Wang W.H., Wen L., Cui X.H., Bao Y.N., Ren H.W., Wang G.Y., Hu W.J., Yuan R.Q. (2026). FXR-mediated transcriptional regulation of KLF11 mitigates contrast-induced acute kidney injury via suppressing JAK2/STAT3 pathway. Int. Immunopharmacol..

[4] Zheng X.Y., Xu Y.J., Wang S.B., Rong C.Y., Zhou F.F., Luo Q. (2026). In-hospital electronic monitoring system approaches to epidemiologic investigation and predictive modeling of contrast-induced acute kidney injury. Ren. Fail..

[5] Sabry S., Ammar M.K., Taeima M., Nassar N., ElFiky A., Saleh A. (2026). Preventing Contrast-Induced Acute Kidney Injury in Egyptian Patients Undergoing Coronary Angiography: A Randomized Controlled Trial. Clin. Drug Investig..

[6] Yang S.C., Zhu S., Zhai X.F., Liu M.X., Zhang P., Fu N.K. (2025). The impact of short-term administration of dapagliflozin on contrast-induced acute kidney injury in patients with type 2 diabetes and renal insufficiency undergoing percutaneous coronary intervention. Front. Med..

[7] Chen S.Y., Scadeng M. (2026). Systematic Review of Intravenous Contrast-Induced Nephropathy and Prophylaxis with Hydration. J. Med. Imaging Radiat. Oncol..

[8] Lin I.C., Tsai W.W., Wu V.C., Pan H.C., Chuang M.H., Chen J.Y. (2025). Saline and N-acetylcysteine-based strategies and other approaches to prevent the risk of CA-AKI: A meta-analysis. Front. Med..

[9] Mehran R., Dangas G.D., Weisbord S.D. (2019). Contrast-Associated Acute Kidney Injury. N. Engl. J. Med..

[10] Deng K., Pei M.X., Li B.B., Yang N.Q., Wang Z.J., Wan X.C., Zhong Z.Y., Yang Z.Y., Chen Y.L. (2024). Signal pathways involved in contrast-induced acute kidney injury. Front. Physiol..

[11] Wu T., Wu X., Cai J., Tang C.Y., Wang X.F., Zeng M.Y., Liu Y.T., Tang Y., Liu Z.W., Meng W. (2026). STING1 exacerbates iodinated contrast-induced acute kidney injury by promoting ferroptosis through chaperone-mediated autophagic degradation of FTH1. Autophagy.

[12] Song S.Y., Su W.Y., Wang H.B., Hong X.C., Xiao Y.L., Zeng X.D. (2025). Advances in therapeutic strategies for acute kidney injury. Life Sci..

[13] Luo Y.F., Long M.Y., Wu X.Q., Zeng L.T. (2025). Targeting ferroptosis: Novel therapeutic approaches and intervention strategies for kidney diseases. Front. Immunol..

[14] Gao Z., Zhang Z.Y., Gu D.Q., Li Y.Q., Zhang K., Dong X.L., Liu L.L., Zhang J.Y., Chen J.M., Wu D.Z. (2022). Hemin mitigates contrast-induced nephropathy by inhibiting ferroptosis via HO-1/Nrf2/GPX4 pathway. Clin. Exp. Pharmacol. Physiol..

[15] Elgendy H.A., Osman A., Farage A.E., Taha M., Abubakr S., Badawy A.M., Ibrahim M.M., Nasr A.N.A., Mahfouz H., Baokbah T.A.S. (2025). Icariin mitigates experimental contrast-induced nephropathy: Mechanistic insights. Tissue Cell.

[16] Yang Q., Ren G.X., Liu M., Peng H.J., Wang W.C., Jiang H.Y., Wei J., Zhu S., Yang H.F., Gu Z.J. (2025). Fullerenol-selexipag suspension alleviates contrast-induced acute kidney injury. Chem. Eng. J..

[17] Tian S.S., Jiang X.X., Tang Y.P., Han T. (2022). *Laminaria japonica* fucoidan ameliorates cyclophosphamide-induced liver and kidney injury possibly by regulating Nrf2/HO-1 and TLR4/NF-κB signaling pathways. J. Sci. Food Agric..

[18] Ben Khelifa H., Dammak M.I., Msehli A., Chebbi R., Ben Said R., Le Cerf D., Majdoub H., Bouraoui A. (2026). Physico-chemical characterization and evaluation of the antioxidant, anti-inflammatory and antinociceptive potential of fucoidan from the Mediterranean seaweed, *Sargassum vulgare*. Algal. Res..

[19] Huang H.Y., Liu Y.P., Xu Z., Zhang D.D., Feng M.M., Zhao T., Zhang L.Y., Li W.J., Li X. (2022). Effect of fucoidan on kidney injury in type 2 diabetic rats based on PI3K/AKT/Nrf2. J. Funct. Foods.

[20] Hamouda H.I., Li T., Shabana S., Hashem A.H., Yin H. (2025). Advances in fucoidan and fucoidan oligosaccharides: Current status, future prospects, and biological applications. Carbohydr. Polym..

[21] Xue M.L., Du R.H., Zhou Y.F., Liu Y.H., Tian Y.J., Xu Y., Yan J.Y., Song P.Z., Wan L., Xu H.S. (2024). Fucoidan supplementation relieved kidney injury and modulated intestinal homeostasis in hyperuricemia mice. J. Agric. Food Chem..

[22] Ren P.F., Liu M., Wei B.Q., Tang Q.J., Wang Y.M., Xue C.H. (2025). Fucoidan exerts antitumor effects by regulating gut microbiota and tryptophan metabolism. Int. J. Biol. Macromol..

[23] Kim H.S., Lee P.C.W., Jin J.-O. (2025). Nasal administration of *Durvillaea antarctica* fucoidan inhibits lung cancer growth in mice through immune activation. Pharmaceuticals.

[24] Dong J.Y., Yang F., Xu Y.Z., Zhao Q.L., Li X.H., Liu T., Tang Y.P. (2025). Exploring the mechanism of kidney injury in mice induced by high-fat diet and polystyrene nanoplastics co-exposure through the kidney-gut axis. J. Agric. Food Chem..

[25] Liu Y.D., He J., Luan W.Y., Xu Y.F., Li Q.Q., Pan L.S., Liu J.G. (2023). A new autophagy-driven protective pathway of Haematococcus astaxanthin against chemical damage in wistar rats with gentamicin-induced acute kidney injury. Algal. Res..

[26] Gui D.K., Huang J.H., Liu W., Guo Y.P., Xiao W.Z., Wang N.S. (2013). Astragaloside IV prevents acute kidney injury in two rodent models by inhibiting oxidative stress and apoptosis pathways. Apoptosis.

[27] Oh H., You J.S., Bae H., Park G.B., Chung Y.E. (2023). Delivery of recombinant sestrin2 ameliorates oxidative stress, mitochondrial damage and renal dysfunction in contrast-induced acute kidney injury. Biochem. Pharmacol..

[28] Venkatachalam S.K., Digala P., Duraisamy N., Santhoshkumar M., Dharmaraj S., Abdi G. (2026). Fucoidan: A promising natural therapeutic agent for protecting human kidney health. Food Hydrocoll. Health.

[29] Chu X.R., Wang X., Feng K.Q., Bi Y.Z., Xin Y.N., Liu S.S. (2025). Fucoidan ameliorates lipid accumulation, oxidative stress, and NF-κB-mediated inflammation by regulating the PI3K/AKT/Nrf2 signaling pathway in a free fatty acid-induced NAFLD spheroid model. Lipids Health Dis..

[30] Liang X.B., Chen Y.H., Zheng Z.J., Zheng Y.H., Wu H.C. (2025). Fucoidan protects against LPS-induced mastitis and enhances the integrity of blood-milk barrier by activating AMPK/Nrf2 and autophagy. Food Sci. Hum. Well..

[31] Lee D., Kim C.-E., Park S.-Y., Kim K.O., Hiep N.T., Lee D., Jang H.-J., Lee J.W., Kang K.S. (2018). Protective effect of *Artemisia argyi* and its flavonoid constituents against contrast-induced cytotoxicity by iodixanol in LLC-PK1 cells. Int. J. Mol. Sci..

[32] Esparham F., Rajabian F., Rahbardar M.G., Razavi B.M., Rad A.K., Amoueian S., Hosseinzadeh H. (2025). Evaluating the effect of rutin on contrast-induced nephropathy in rats. Avicenna J. Phytomed..

[33] Li Y., Huang M., Guo X., Liu X., Wang J. (2026). Integration of network pharmacology and transcriptomics to reveal the ROS/NLRP3/Caspase-1/GSDMD-mediated mechanism of baicalin in alleviating contrast-induced acute kidney injury. Naunyn-Schmiedebergs Arch. Pharmacol..

[34] Tian J., Xiao F., Xu Y.Z., Chen Y., He C.L., Jiang S., Tang Y.P. (2026). Combined metabolomics and gut microbiota analysis reveals the protective effects of α-monoglucosyl rutin against cyclophosphamide-induced intestinal injury. Int. Immunopharmacol..

[35] Jiang L., Xue T.C., Zhang H., Zhang Q.Y., Deng H.L. (2026). Effect of traditional Chinese medicine on disturbances of untargeted metabolomics in ulcerative colitis. J. Ethnopharmacol..

[36] He Q.Y., Ye B.B., Li H.Q., Pan Y.Q., Du Y., Yang K. (2025). Analysis of potential biomarkers for diabetic kidney disease and non-diabetic kidney disease based on urinary metabolomics analysis. BMC Nephrol..

[37] Zhang B., Zeng M., Wang Y., Li M., Wu Y., Xu R., Zhang Q., Jia J., Huang Y., Zheng X. (2022). Oleic acid alleviates LPS-induced acute kidney injury by restraining inflammation and oxidative stress via the Ras/MAPKs/PPAR-γ signaling pathway. Phytomedicine.

[38] Li X., Wang S., Liu Z., Jia Y., Su X., Yang C., Ge Q., Zhao L., Zhao R. (2026). Targeted metabolomics of nucleotide intermediates for biomarker discovery in acute kidney injury. Anal. Methods.

[39] Qiu S., Xie D., Guo S., Wang Z., Cai Y., Wang X., Hu Z., Wang S., Lin C., Yao H. (2026). MAPK14/SLC7A11/GPX4 axis dysregulation drives podocyte ferroptosis via mediating glycerophospholipid metabolism. Cell Death Discov..

[40] Shu L., Fu H.J., Pi A.W., Feng Y.L., Dong H., Si C.J., Li S.T., Zhu F.Y., Zheng P.F., Zhu Q. (2024). Protective effect of andrographolide against ulcerative colitis by activating Nrf2/HO-1 mediated antioxidant response. Front. Pharmacol..

[41] Wei X., Luo Y.L., Yuan D.J., Li D., Nong Y.X., Wu B.L., Qin X.J. (2025). Effect of the Nrf2/HO-1 pathway on aluminum-induced liver injury. Ecotox. Environ. Saf..

[42] Li X., Sun Y.X., Tjahjono A.W., Wei Y., Li X., Zheng Q.H., Qi W.C., Liang F.R. (2025). Acupuncture attenuates myocardial ischemia/reperfusion injury-induced ferroptosis via the Nrf2/HO-1 pathway. Chin. Med..

[43] Zhu Z.Q., Li J., Song Z.Y., Li T.L., Li Z.P., Gong X.Z. (2024). Tetramethylpyrazine attenuates renal tubular epithelial cell ferroptosis in contrast-induced nephropathy by inhibiting transferrin receptor and intracellular reactive oxygen species. Clin. Sci..

[44] Wang Y.Z., Han J.W., Zhan S.F., Guo C.Y., Yin S.N., Zhan L., Zhou Q.Y., Liu R.Y., Yan H., Wang X.Y. (2024). Fucoidan alleviates doxorubicin-induced cardiotoxicity by inhibiting ferroptosis via Nrf2/GPX4 pathway. Int. J. Biol. Macromol..

[45] Li J.J., Xu W.Q., Li Y.Y., Guo C.Y., Xu X.F. (2023). Fucoidan Ameliorates Ferroptosis in ischemia-reperfusion-induced Liver Injury through Nrf2/HO-1/GPX4 activation. J. Clin. Transl. Hepatol..

[46] Sakai C., Abe S., Kouzuki M., Shimohiro H., Ota Y., Sakinada H., Takeuchi T., Okura T., Kasagi T., Hanaki K. (2019). A randomized placebo-controlled trial of an oral preparation of high molecular weight fucoidan in patients with type 2 diabetes with evaluation of taste sensitivity. Yonago Acta Med..

[47] Tomori M., Nagamine T., Miyamoto T., Iha M. (2021). Effects of ingesting fucoidan derived from cladosiphon okamuranus tokida on human NK cells: A randomized, double-blind, parallel-group, placebo-controlled pilot study. Mar. Drugs.

[48] Abe S., Hiramatsu K., Ichikawa O., Kawamoto H., Kasagi T., Miki Y., Kimura T., Ikeda T. (2013). Safety evaluation of excessive ingestion of mozuku fucoidan in human. J. Food Sci..

[49] Agmon Y., Peleg H., Greenfeld Z., Rosen S., Brezis M. (1994). Nitric oxide and prostanoids protect the renal outer medulla from radiocontrast toxicity in the rat. J. Clin. Investig..

[50] Li F.H., Tian J., Xu Y.Z., Yi W.T., Chen Q., Wang J.T., Jiang S., Tang Y.P., Han T. (2025). Physicochemical characteristics and therapeutic mechanisms of *Sargassum horneri*-derived soluble dietary fiber in cyclophosphamide-induced intestinal damage via gut microbiota and metabolic modulation. Food Biosci..

